# Identification of a novel cuproptosis‐related gene signature for multiple myeloma diagnosis

**DOI:** 10.1002/iid3.1058

**Published:** 2023-11-07

**Authors:** Yidong Zhu, Shuaikang Chang, Jun Liu, Bo Wang

**Affiliations:** ^1^ Department of Traditional Chinese Medicine, Shanghai Tenth People's Hospital Tongji University School of Medicine Shanghai China; ^2^ Department of Hematology, Shanghai East Hospital Tongji University School of Medicine Shanghai China; ^3^ Department of Endocrinology, Yangpu Hospital Tongji University School of Medicine Shanghai China

**Keywords:** biomarker, cuproptosis, LASSO, multiple myeloma, SVM‐RFE, tumor immunity

## Abstract

**Background:**

Multiple myeloma (MM) ranks second among the most prevalent hematological malignancies. Recent studies have unearthed the promise of cuproptosis as a novel therapeutic intervention for cancer. However, no research has unveiled the particular roles of cuproptosis‐related genes (CRGs) in the prediction of MM diagnosis.

**Methods:**

Microarray data and clinical characteristics of MM patients were obtained from the Gene Expression Omnibus (GEO) database. Differentially expressed gene analysis, least absolute shrinkage and selection operator (LASSO) and support vector machine‐recursive feature elimination (SVM‐RFE) algorithms were applied to identify potential signature genes for MM diagnosis. Predictive performance was further assessed by receiver operating characteristic (ROC) curves, nomogram analysis, and external data sets. Functional enrichment analysis was performed to elucidate the involved mechanisms. Finally, the expression of the identified genes was validated by quantitative real‐time polymerase chain reaction (qRT‐PCR) in MM cell samples.

**Results:**

The optimal gene signature was identified using LASSO and SVM‐RFE algorithms based on the differentially expressed CRGs: *ATP7A*, *FDX1*, *PDHA1*, *PDHB*, *MTF1*, *CDKN2A*, and *DLST*. Our gene signature‐based nomogram revealed a high degree of accuracy in predicting MM diagnosis. ROC curves showed the signature had dependable predictive ability across all data sets, with area under the curve values exceeding 0.80. Additionally, functional enrichment analysis suggested significant associations between the signature genes and immune‐related pathways. The expression of the genes was validated in MM cells, indicating the robustness of these findings.

**Conclusion:**

We discovered and validated a novel CRG signature with strong predictive capability for diagnosing MM, potentially implicated in MM pathogenesis and progression through immune‐related pathways.

## Graphical abstract

We identified a novel signature associated with cuproptosis‐related genes, which exhibited a strong diagnostic value for multiple myeloma (MM). Our study highlights the involvement of cuproptosis‐related genes in the diagnosis of MM and investigates the underlying molecular mechanisms, offering novel insights into the pathogenesis and progression of MM.

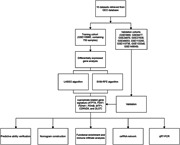

## 1 INTRODUCTION

Multiple myeloma (MM), which is the second most common hematological malignancy in high‐income countries, is characterized by the uncontrolled accumulation of monoclonal plasma cells in the bone marrow.^1^, ^2^ Despite enormous progress in the treatment of MM which has improved the rates of response and survival over the last decade, relapse still occurs, and the cure remains elusive.^3^, ^4^, ^5^, ^6^ The symptoms reported by MM patients on presentation are usually nonspecific and may have already been present for an extended period.^3^, ^7^ Asymptomatic patients present with only laboratory abnormalities, such as anemia, renal disease, and elevated protein levels.^8^, ^9^ Currently, no single test can reliably diagnose or monitor all MM patients. The diagnosis of MM is mainly based on the results of clinical chemistry tests, cytogenetic analysis of bone marrow, and radiological examinations for the detection of bone changes.^7^, ^10^, ^11^, ^12^ Early diagnosis and subsequent management of MM could improve patients' quality of life and reduce the symptom burden and serious complications of this severe disease.^1^, ^3^, ^13^ Therefore, the identification and establishment of new, reliable, and sensitive diagnostic biomarkers to detect the early stages of the disease is crucial for effective malignancy treatment.

Copper is one of the essential trace elements in the human body, which can also be noxious when its concentration exceed a certain threshold.^14^ “Cuproptosis” is a term coined to describe a recently discovered mode of mitochondrial cell death triggered by copper.^15^ Intracellular copper directly binds to lipoylated components of the tricarboxylic acid (TCA) cycle, which induces the aggregation of mitochondrial lipoylated proteins and destabilization of iron‐sulfur cluster proteins, resulting in proteotoxic stress and ultimately cell death.^15^ The dyshomeostasis of copper has been associated with MM. For example, several studies found that a high copper level in the serum of MM patients.^16^, ^17^, ^18^ Besides, a complex of copper and disulfiram was established to exert a significant inhibitory effect on the growth of myeloma cell lines.^19^, ^20^ Previous reports have described the functional roles of cuproptosis‐related genes (CRGs) in cancer development. For instance, CRGs were used to predict clinical outcome and immune response in hematological malignancies, including acute myeloid leukemia^21^, ^22^ and diffuse large B‐cell lymphoma.^23^ The results of recent studies have emphasized the potential utility of CRGs in the development of prognostic models for MM.^24^, ^25^ These findings indicate the existence of an association between CRGs and MM. However, the primary focus of these studies has been placed on the use of CRGs for the prediction of MM prognosis rather than on its diagnosis.

Considering the significance of early detection in identifying asymptomatic individuals who would benefit from timely intervention, in this study, we aimed to utilize CRGs for MM diagnostic prediction. We identified a CRG signature for MM diagnosis by analysis of differentially expressed genes, least absolute shrinkage and selection operator (LASSO), and support vector machine‐recursive feature elimination (SVM‐RFE) algorithms. Then, we validated our findings using nine external data sets. Furthermore, we conducted functional enrichment analysis to investigate the potential mechanisms. Additionally, the expression of the identified genes in MM cell samples was validated using quantitative real‐time polymerase chain reaction (qRT‐PCR). Our study discovered a novel role of CRGs in MM diagnosis and explored the underlying molecular mechanisms. Our findings are expected to contribute to more effective decision‐making processes in establishing and implementing strategies for MM diagnosis and treatment.

## 2 MATERIALS AND METHODS

### 2.1 Data acquisition

Ten data sets (Supporting Information: Table S1) comprising MM and control samples, namely GSE5900, GSE6477, GSE24870, GSE27838, GSE46053, GSE113295, GSE113736, GSE118985, GSE133346, and GSE146649, were obtained from the Gene Expression Omnibus database (GEO, https://www.ncbi.nlm.nih.gov/geo/). The raw data of all analyzed data sets were normalized to remove batch effects. The GSE118985 data set served as the training cohort for subsequent analyses, whereas the GSE5900, GSE6477, GSE24870, GSE27838, GSE46053, GSE113295, GSE113736, GSE133346, and GSE146649 data sets were selected to serve as diverse external validation cohorts for result verification. Based on our literature review,^15^, ^26^, ^27^, ^28^, ^29^ a total number of 19 CRGs (Supporting Information: Table S2) were subjected to analysis.

### 2.2 Identification of differentially expressed CRGs

The expression data of CRGs in MM and control samples from the training cohort were extracted and subjected to differential expression analysis with the “LIMMA” package. Differentially expressed CRGs were required to meet the criterion of adjusted *p* < .05. The heatmap of the differentially expressed CRGs was visualized by the “pheatmap” package in R software.

### 2.3 Identification of the optimal gene signature

LASSO is a regressive arithmetic method that ameliorates forecast accurateness for high‐dimensional data and prevents overfitting during modeling.^30^, ^31^ Moreover, SVM‐RFE algorithm can effectively extract the information of the most relevant features through nonlinear kernels to screen out the best combination of variables.^32^ The optimal panel of predictive signature genes was identified by selecting the overlapping genes obtained from the LASSO and SVM‐RFE methods based on the differentially expressed CRGs. Additionally, receiver operating characteristic (ROC) curves were constructed to assess the accuracy and specificity of these genes in diagnosing MM. The results of these analyses were visualized by the “glmnet,” “e1071,” “VennDiagram,” and “pROC” packages.

### 2.4 Nomogram construction

A nomogram was constructed based on the obtained gene signature to forecast the risk of disease. The predictive ability of the established model was evaluated using the calibration curve and decision curve analysis (DCA). The “rms” and “rmda” packages were employed to perform these analyses and visualize their results.

### 2.5 Validation of the gene signature

The ability of the gene signature to differentiate between MM and controls was assessed in nine validation cohorts: GSE5900, GSE6477, GSE24870, GSE27838, GSE46053, GSE113295, GSE113736, GSE133346, and GSE146649, using ROC curve analysis. Visualization of the results was performed using the “glmnet” and “pROC” packages.

### 2.6 Functional enrichment analysis

Gene set enrichment analysis (GSEA) software was employed to explore the potential mechanisms by identifying significantly enriched pathways for each signature gene using the gene set (Kegg.v7.4.symbols.gmt). Moreover, the CIBERSORT algorithm was applied to compare the levels of 22 distinct infiltrating immune cell types between the MM and control individuals. We performed Spearman correlation analysis to assess the correlation between the signature genes and the infiltrating immune cells. The results of these analyses were visualized by the “clusterProfiler,” “enrichplot,” “DOSE,” “org.Hs.eg.db,” “preprocessCore”, “ggpubr,” “tidyverse,” “ggplot2,” and “reshape2” packages.

### 2.7 Construction of a competitive endogenous RNA (ceRNA) network

A ceRNA network was built by exploring the interaction relationships among messenger RNAs (mRNAs), microRNAs (miRNAs), and long noncoding RNAs (lncRNAs). The mRNA and miRNA interaction pairs were predicted by the intersection of miRDB (https://www.mirdb.org/), TargetScan (https://www.targetscan.org/vert_80/), and miRanda (www.microrna.org) databases based on the signature genes. Subsequently, we searched for potential lncRNAs interactions with miRNAs on the spongeScan database (http://spongescan.rc.ufl.edu/). Visualization of the mRNA–miRNA–lncRNA ceRNA network was performed with Cytoscape software (version 3.7.0).

### 2.8 qRT‐PCR

MM cell line H929 cells were commercially obtained from the American Type Culture Collection (ATCC). These cells were cultured in RPMI‐1640 medium (Gibco) supplemented with 10% fetal bovine serum (Gibco) and 1% penicillin–streptomycin (Gibco). Peripheral blood mononuclear cells (PBMCs) were isolated from healthy donors using Ficoll‐Hypaque density gradient centrifugation after obtaining informed consent. This study was approved by the Ethics Committee of Shanghai Yangpu Hospital (LL‐009). All cells were incubated at 37°C in a humidified atmosphere with 5% carbon dioxide. Total RNA was extracted using TRIzol reagent (Invitrogen), following the manufacturer's protocol. Subsequently, complementary DNA was reverse‐transcribed from 1 μg of total RNA using the PrimeScript™ RT Reagent Kit (Takara). qRT‐PCR was performed in 96‐well plates using the SYBR Green PCR Master Mix (KAPA) and the Applied Biosystems 7900HT Fast Real‐Time PCR System (Thermo Fisher Scientific). Gene expression levels were determined using the $2^{-∆∆C_{t}}$ algorithm, with ACTIN serving as the internal control. The primers utilized for qRT‐PCR are presented in Table 1.

**Table 1:** Primers used for qRT‐PCR.

Gene name	Strand	5′–3′
*ATP7A*	Forward	TGCCATCCTCCATGCTTTCA
	Reverse	ACTGGCTGTGTTCAACTGCT
*FDX1*	Forward	AAGGGAATGAGCAGGCTGAC
	Reverse	CACTCCTGGGACTGACACAC
*PDHA1*	Forward	GACTCGGGAACAAGAAGGCA
	Reverse	GCTGGGAAAAAGCCAAGGTG
*PDHB*	Forward	ATGGTGCCTCAGCAGGTGTA
	Reverse	AGCACCACCACTGGATTGTT
*MTF1*	Forward	TGCTTAGCACCAGGGATTGG
	Reverse	CTTCCCTCAATGCCCAACCT
*CDKN2A*	Forward	CCACCCCGCTTTCGTAGTT
	Reverse	AGTGAAAAGGCAGAAGCGGT
*DLST*	Forward	TCTAGGAGGATGCTGTGCCT
	Reverse	TGCCTGTGTTCAATCCCTCC

Abbreviation: qRT‐PCR, quantitative real‐time polymerase chain reaction.

### 2.9 Statistical analysis

Data analysis was conducted using R software (version 4.3.0) and GraphPad Prism software (version 8.0.1). For quantitative variables, differences between groups with normally distributed variables were analyzed using Student's *t* test, whereas Wilcoxon test was employed for skewed data. A two‐sided *p* < .05 was considered to indicate a statistically significant difference. The significance levels are denoted as follows: *p < .05, **p < .01, and ***p < .001.

## 3 RESULTS

### 3.1 Identification of differentially expressed CRGs

**Figure 1:**
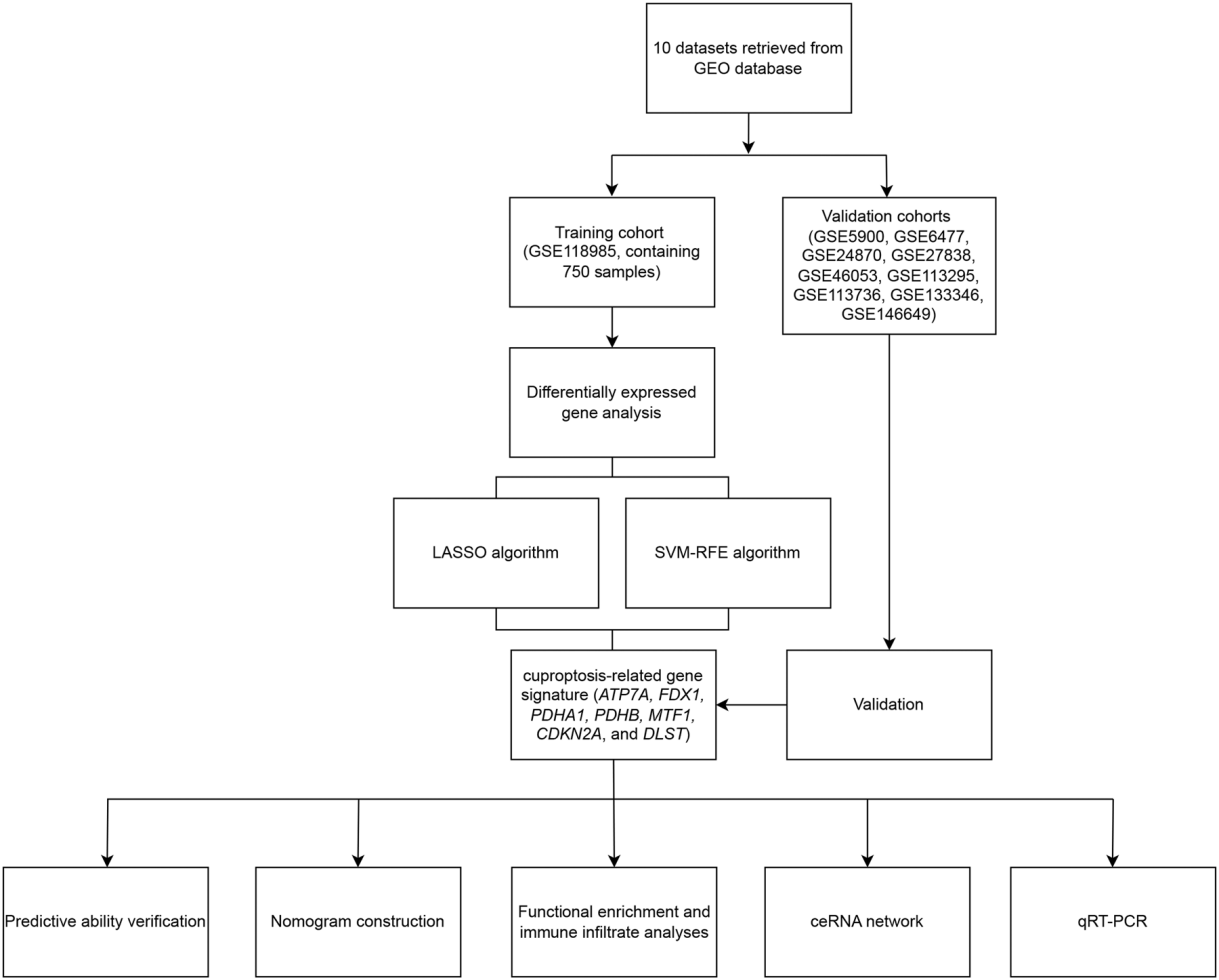
Flowchart of this study. GEO, Gene Expression Omnibus; LASSO, least absolute shrinkage and selection operator; qRT‐PCR, quantitative real‐time polymerase chain reaction; SVM‐RFE, support vector machine‐recursive feature elimination.

**Figure 2:**
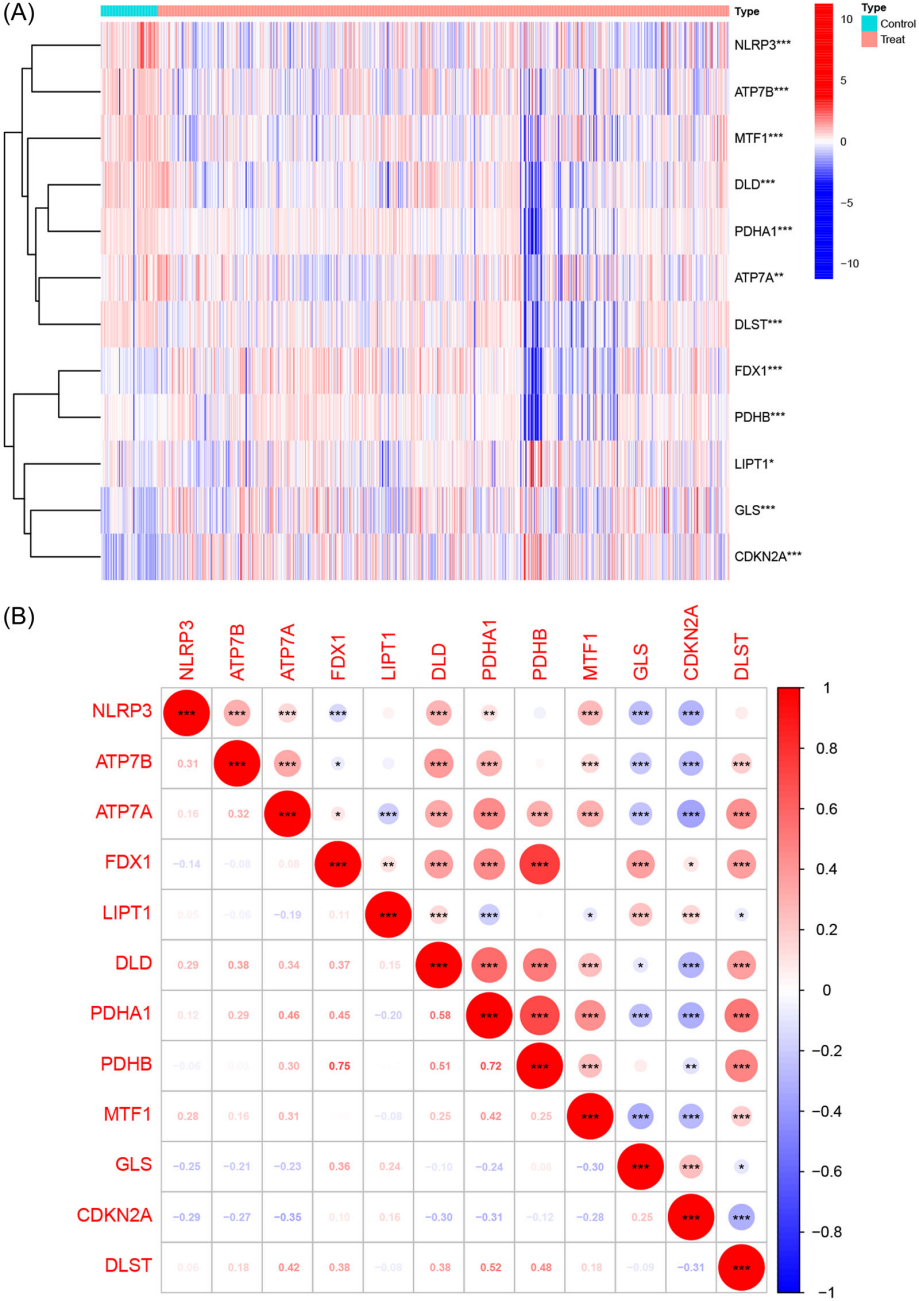
Identification of differentially expressed cuproptosis‐related genes (CRGs). (A) Heatmap of differentially expressed CRGs; (B) correlation plot of differentially expressed CRGs.

Figure 1 depicts the flowchart of gene signature identification and validation, nomogram construction, and the subsequent analyses conducted in this study. Based on the criteria applied to the training cohort, 12 CRGs were identified as differentially expressed genes between the MM and control samples (Figure 2A, Supporting Information: Table S3). Compared with the normal controls, the expression levels of *FDX1*, *LIPT1*, *PDHB*, *GLS*, and *CDKN2A* were upregulated, whereas the expression levels of *NLRP3*, *ATP7B*, *ATP7A*, *DLD*, *PDHA1*, *MTF1*, and *DLST* were downregulated in the MM samples. Besides, the identified genes had significant synergistic effects, which are visualized in Figure 2B to display their interactions and interrelationships.

### 3.2 Identification of the optimal gene signature

**Figure 3:**
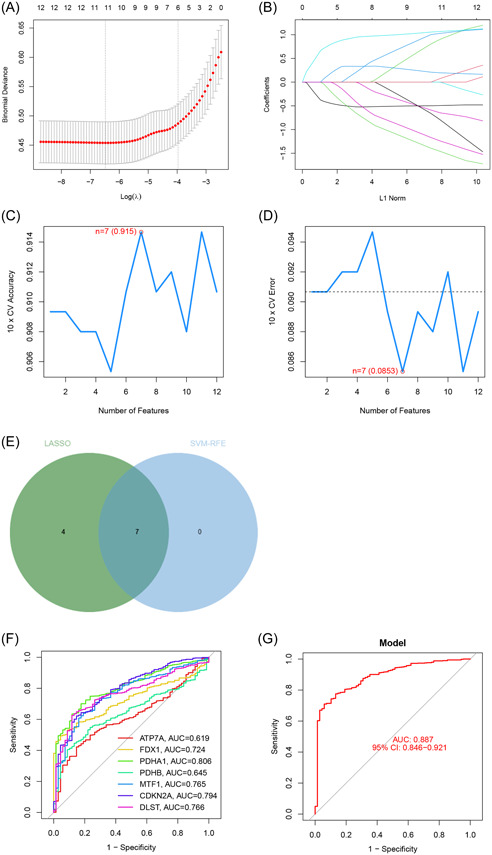
Identification of the optimal gene signature. (A, B) Least absolute shrinkage and selection operator (LASSO) algorithm to identify the optimal genes; (C, D) support vector machine‐recursive feature elimination (SVM‐RFE) algorithm for the identification of the optimal combination of feature genes; (E) overlapped genes based on the intersections of the LASSO and SVM‐RFE algorithms; (F) receiver operating characteristic (ROC) curves of the signature genes in the training cohort; (G) ROC curve of the gene signature‐based model in the training cohort. AUC, area under the curve; CI, confidence interval.

Different machine learning algorithms were employed to identify the optimal gene signature for MM diagnosis based on the differentially expressed CRGs. The LASSO algorithm was used to extract a total number of 11 genes (Figure 3A,B). The SVM‐RFE algorithm was applied to filter 7 genes as the optimal feature gene combination (Figure 3C,D). As a result, seven overlapped genes (*ATP7A*, *FDX1*, *PDHA1*, *PDHB*, *MTF1*, *CDKN2A*, and *DLST*) were identified as the optimal CRG signature for diagnostic prediction in MM patients (Figure 3E). Moreover, ROC curves were generated to establish the diagnostic values of the signature genes for MM. As can be seen in Figure 3G, the area under the curve (AUC) values for all genes exceeded 0.6. We also constructed a logistic regression model utilizing the obtained gene signature for diagnostic prediction, with the AUC value was 0.887 (Figure 3G), indicating a high predictive value for MM. The results revealed that the gene signature‐based model outperformed the individual genes in terms of diagnostic value for MM.

### 3.3 Nomogram construction

**Figure 4:**
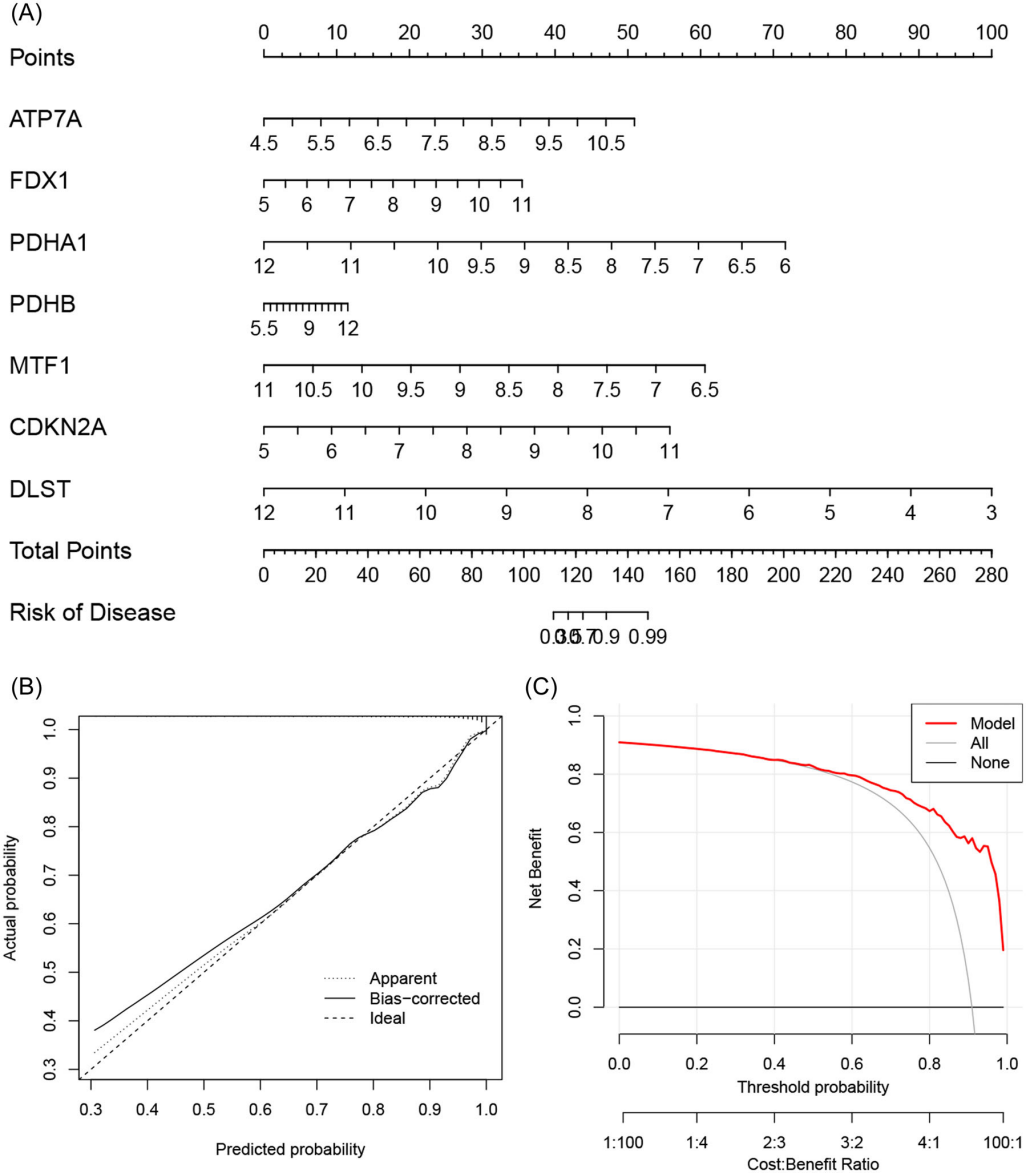
Nomogram construction. (A) multiple myeloma risk prediction nomogram; (B) calibration curve; (C) decision curve analysis.

We developed a nomogram utilizing the identified gene signature to predict the risk of MM (Figure 4A). The calibration curve showed the ideal agreement between the observed practical outcomes and the predicted risk probabilities (Figure 4B). DCA revealed that the constructed nomogram achieved a satisfactory benefit for clinical decision‐making (Figure 4C).

### 3.4 Validation of the gene signature

**Figure 5:**
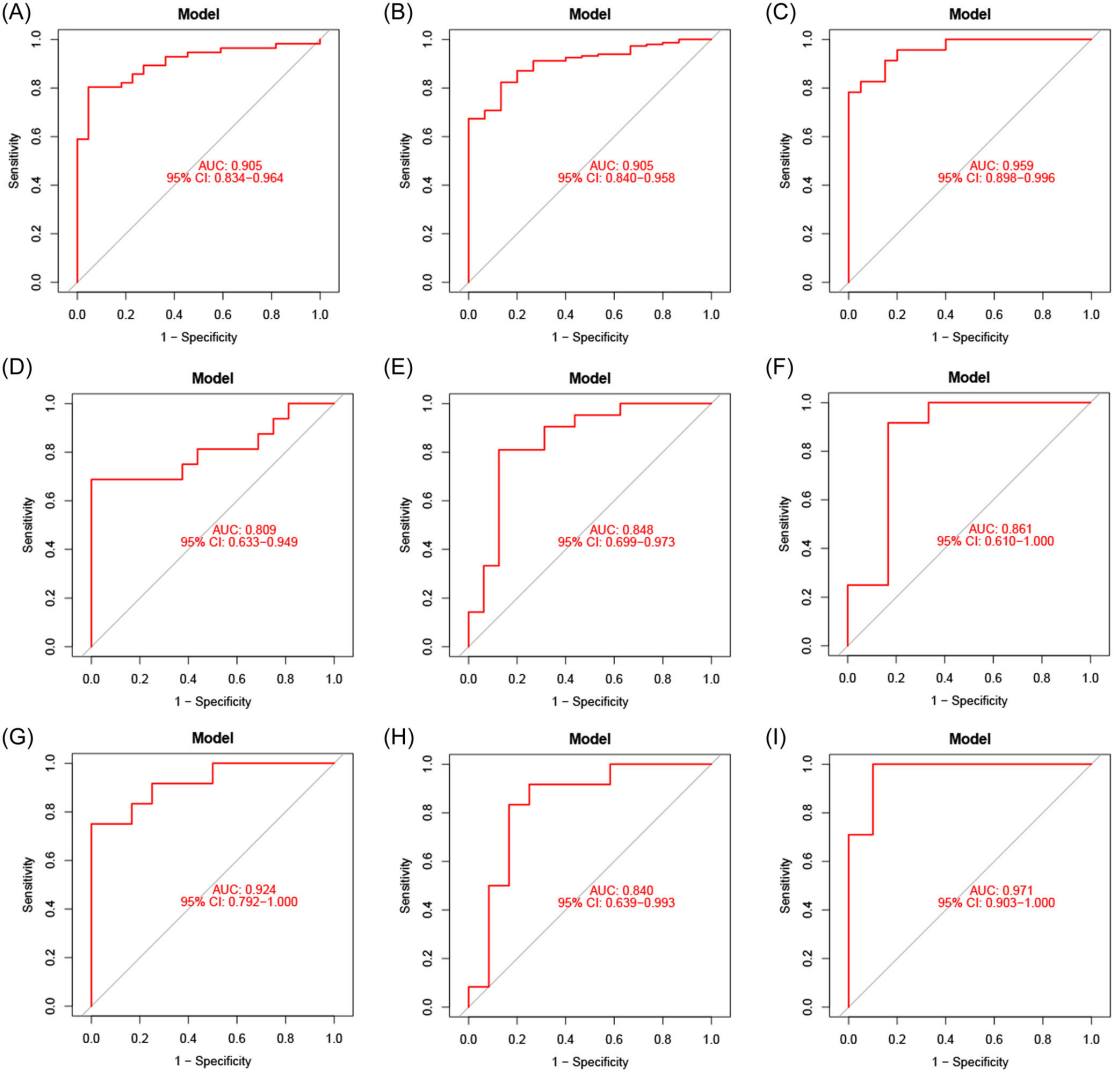
Validation of the gene signature. Receiver operating characteristic curves of the gene signature in the GSE5900 (A), GSE6477 (B), GSE24870 (C), GSE27838 (D), GSE46053 (E), GSE113295 (F), GSE113736 (G), GSE133346 (H), and GSE146649 (I) data sets, respectively.

We validated our gene signature‐based model using nine external data sets containing MM and control samples. Satisfactory model performance was achieved as determined by the ROC curves with AUC values of 0.905, 0.905, 0.959, 0.809, 0.848, 0.861, 0.924, 0.840, and 0.971 for the GSE5900, GSE6477, GSE24870, GSE27838, GSE46053, GSE113295, GSE113736, GSE133346, and GSE146649 data sets, respectively (Figure 5A–I). Therefore, the gene signature‐based model was confirmed to have a strong MM diagnostic value in all the training and validation cohorts, with AUC values consistently exceeding 0.80.

### 3.5 Functional enrichment analysis

**Figure 6:**
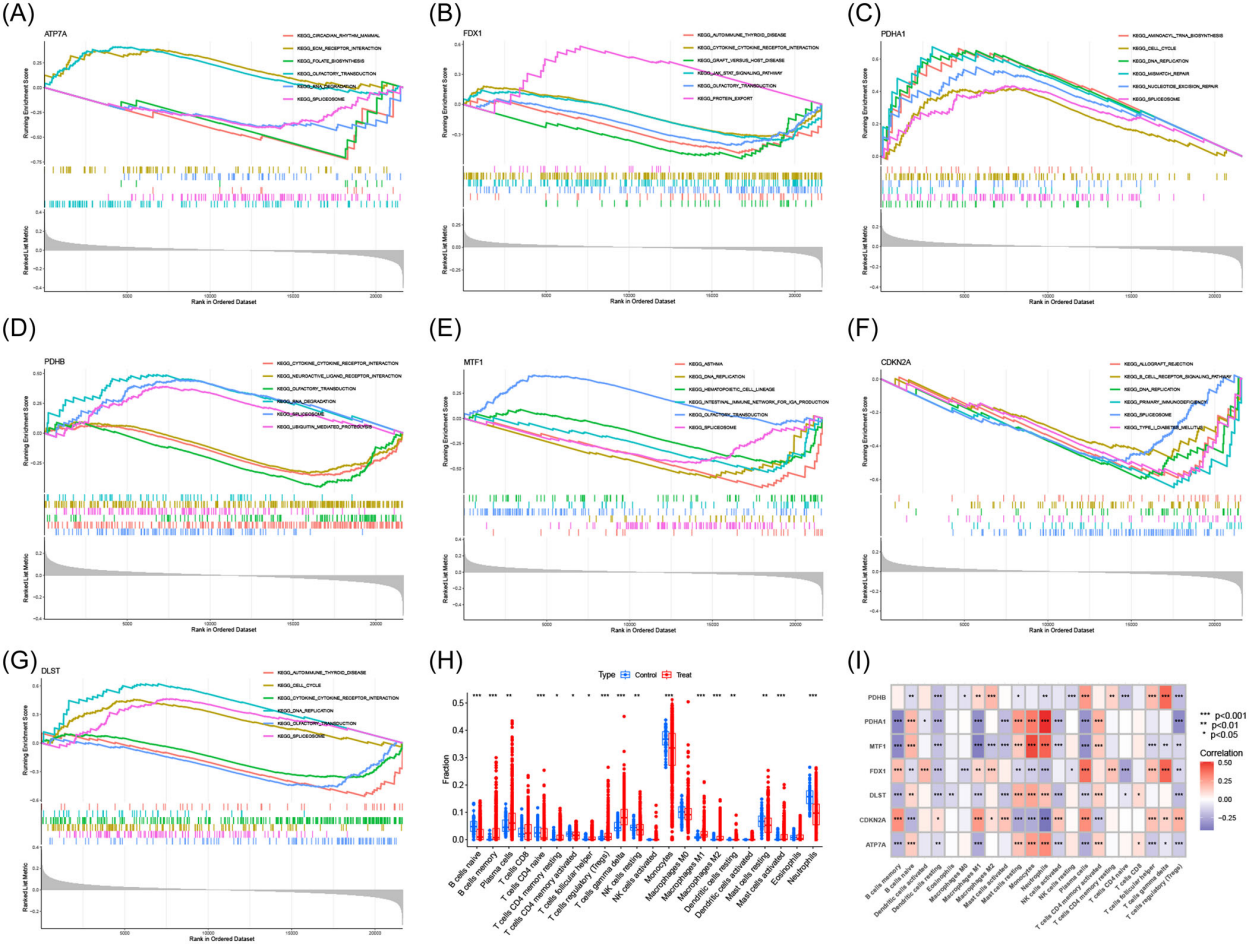
Functional enrichment analysis. (A‐G) Gene set enrichment analysis on enriched pathways of *ATP7A* (A), *FDX1* (B), *PDHA1* (C), *PDHB* (D), *MTF1* (E), *CDKN2A* (F), and *DLST* (G); (H) Boxplot of infiltrating immune cells; (I) Heatmap of correlations between genes and infiltrating immune cells.

The top six pathways enriched for each signature gene are shown in Figure 6A–G. Our findings revealed the enrichment of these genes in immune‐related pathways (e.g., B‐cell receptor signaling pathway, cytokine–cytokine receptor interaction, and intestinal immune network for immunoglobulin A production) and immune‐related diseases (e.g., primary immunodeficiency, autoimmune thyroid disease, and graft‐vs‐host disease). The results of the CIBERSORT algorithm showed significant differences in 17 of 22 infiltrating immune cells between the MM and control samples (Figure 6H). Additionally, our correlation analysis revealed significant associations between the signature genes and the infiltrating immune cells (Figure 6I).

### 3.6 Construction of a ceRNA network

**Figure 7:**
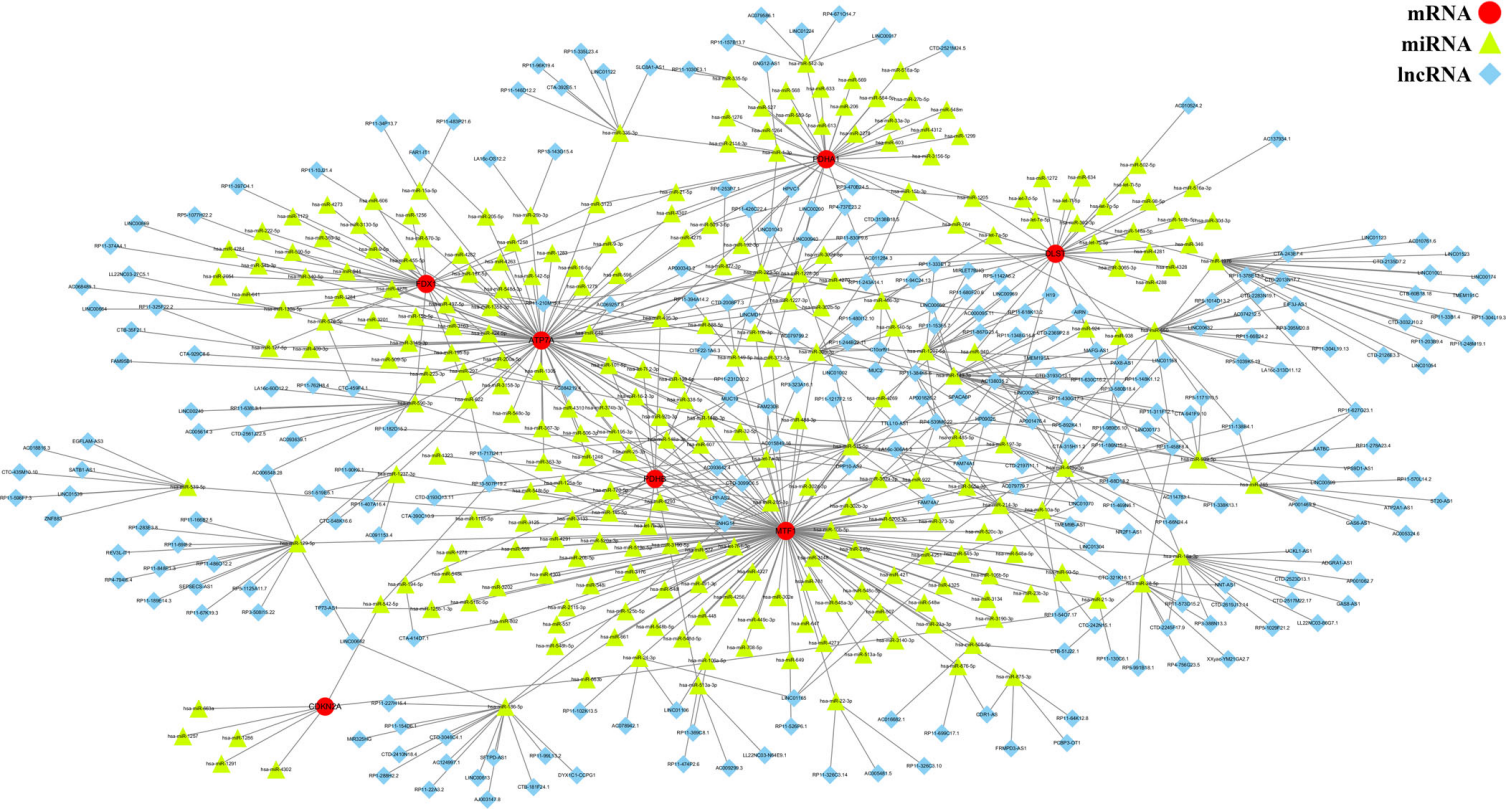
A competitive endogenous RNA network based on the identified biomarkers. mRNA, messenger RNA; miRNA, microRNA; lnRNA, long noncoding RNA.

A ceRNA network was constructed using miRDB, TargetScan, miRanda, and spongeScan databases to investigate the regulation of the signature genes (Figure 7). The network consisted of 524 nodes (including 7 mRNAs, 257 miRNAs, and 260 lncRNAs) and 674 edges (Supporting Information: Table S4).

### 3.7 qRT‐PCR

**Figure 8:**
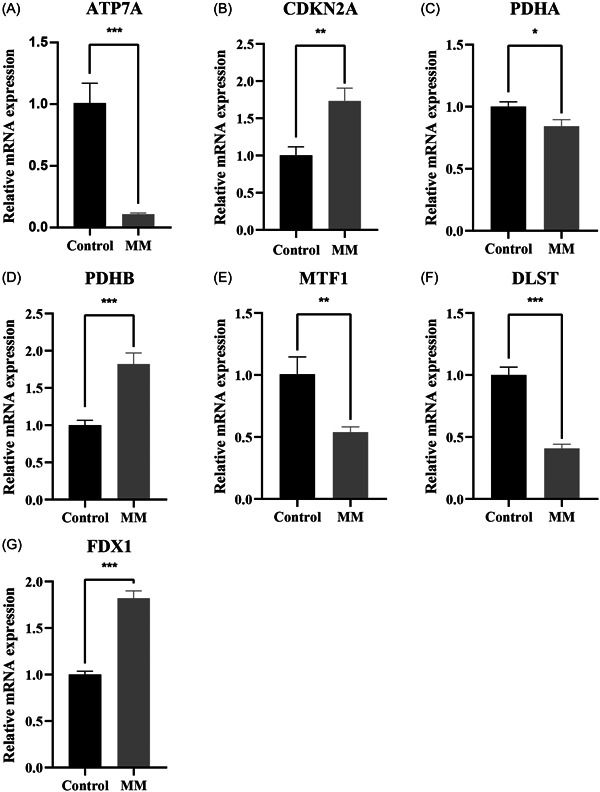
Quantitative real‐time polymerase chain reaction analysis. (A) *ATP7A*; (B) *CDKN2A*; (C) *PDHA1*; (D) *PDHB*; (E) *MTF1*; (F) *DLST*; (G) *FDX1*. *p < .05; **p < .01; ***p < .001. MM, multiple myeloma; mRNA, messenger RNA.

Subsequently, qRT‐PCR was used to validate the expression of the identified genes in the H929 and control cells. Expectedly, the expression levels of *ATP7A*, *PDHA1*, *MTF1*, and *DLST* in the MM samples were significantly lower than those in the control group, whereas the levels of *FDX1*, *PDHB*, and *CDKN2A* were significantly higher in the MM samples (Figure 8).

## 4 DISCUSSION

It is worth noting that previous studies have reported the utilization of CRGs for prognostic prediction in MM.^24^, ^25^ However, given the significance of early diagnosis and effective management for improving patients' quality of life and reducing the burden of symptoms and complications, an exploration of the potential impact of CRGs on predicting the diagnosis of MM is imperative. The main discovery in this study is the successful identification and validation of a gene signature associated with CRGs for MM diagnosis, which was achieved by differentially expressed gene analysis, LASSO, and SVM‐RFE algorithms. This signature identification can facilitate decision‐making on the establishment and implementation of diagnostic strategies for MM in clinical practice.

In this study, 12 of the 19 CRGs were identified as differentially expressed genes between the MM and control samples, underscoring the critical role of CRGs in the development and progression of MM. Leveraging these differentially expressed CRGs, we employed LASSO and SVM‐RFE algorithms to screen the feature variables and construct an optimal diagnostic model. A total number of 11 and 7 genes were extracted using the LASSO and SVM‐RFE algorithms, respectively. Ultimately, a gene signature consisting of seven overlapping genes (*ATP7A*, *FDX1*, *PDHA1*, *PDHB*, *MTF1*, *CDKN2A*, and *DLST*) was identified as the optimal cuproptosis‐related signature for predicting MM diagnosis, with an AUC value of 0.887 in the training cohort. Additionally, the nomogram based on this gene model had high accuracy in predicting MM diagnosis, suggesting that the developed model may serve as a powerful diagnostic tool for MM. Validating the developed gene signature was performed on nine external data sets, confirming its robustness with consistently exceeding AUC values of 0.80. The identified signature genes have various roles. For instance, *ATP7A* functions as an ATP‐driven copper ion pump, thus playing a crucial role in maintaining intracellular copper ion homeostasis.^33^, ^34^, ^35^ *FDX1* is essential for the synthesis of various steroid hormones.^36^, ^37^ *PDHA1* and *PDHB* catalyze the conversion of pyruvate to acetyl‐coenzyme A (CoA) and carbon dioxide, linking the glycolytic pathway to the tricarboxylic cycle.^38^, ^39^, ^40^ *MTF1* acts as a transcription factor that induces the expression of metallothioneins and other genes involved in metal homeostasis in response to heavy metals.^41^ *CDKN2A* was found to be capable of inducing cell cycle arrest in G1 and G2 phases and to be involved in the regulation of autophagy and caspase‐independent cell death.^42^, ^43^, ^44^ *DLST*, a mitochondrial protein belonging to the 2‐oxoacid dehydrogenase family, catalyzes the conversion of 2‐oxoglutarate to succinyl‐CoA and carbon dioxide.^45^, ^46^ It is noteworthy that allelic loss of *CDKN2A* has previously been reported in MM patients.^47^ ln addition, the differential expression of the identified genes was validated in MM cell samples, increasing the robustness of our findings. However, the specific roles of the signature genes in the diagnosis of MM are not yet fully understood.

Functional enrichment analysis was used to investigate the potential mechanisms by which the signature genes contribute to the pathogenesis and progression of MM. GSEA revealed significant enrichment of immune‐related pathways and diseases among the signature genes. Therefore, the CIBERSORT algorithm was employed to assess the disparities in the infiltration of 22 immune cells between the MM and control samples. The results showed that 17 of 22 infiltrating immune cells had significant differences. Among these, the MM group showed significant upregulation of regulatory T cells (Tregs) and mast cells. Tregs are a diverse population of CD4^+^ T cells with suppressive functions crucial in self‐tolerance with different origins, phenotypes, and subtypes.^48^ Earlier studies showed that Tregs may be recruited and exploited by various tumor cells to evade immunosurveillance and eliminate protective antitumor immunity.^49^, ^50^ Increased Tregs were detected in the peripheral blood and bone marrow of MM patients.^51^, ^52^, ^53^, ^54^, ^55^ Besides, cytokines such as tumor necrosis factor‐alpha and interleukin‐10 secreted by mast cells are essential in promoting the immune tolerance mediated by Tregs which contribute to immune suppression and tumor promotion.^56^, ^57^ Additionally, the vascular endothelial growth factor (VEGF) released by mast cells causes an angiogenic response and promotes tumor angiogenesis, which plays vital biological roles in the development of neoplastic disorders.^58^, ^59^ Several studies have evidenced that the degree of mast cell infiltration parallels the severity of MM.^60^, ^61^, ^62^ Additionally, the correlations between the identified genes and the infiltrating immune cells were calculated, revealing notable interactions between the signature genes and tumor immunity. Accumulating evidence has confirmed that the pathogenesis and evolution of MM is associated with loss of immune control, which reflects changes in the immune cells and suppressive bone marrow microenvironment.^63^, ^64^, ^65^ These previous results are in agreement with the findings of the present investigation. Thus, it is reasonable to infer that the identified genes may support the nosogenesis and progression of MM by suppressing the immune response through immune‐related pathways. Finally, gene regulation by the mRNA–miRNA–lncRNA ceRNA network could facilitate further exploration of the molecular mechanisms of MM.

In this study, we successfully identified and validated a novel signature consisting of CRGs, showcasing its potential as a promising biomarker for MM diagnosis. Importantly, the gene signature revealed AUC values exceeding 0.80 in all the data sets, solidifying the robust predictive capacity of our model. Moreover, the expression patterns of the identified genes remained consistent in the MM cell samples, further bolstering the potential for effective clinical application. Of note, the results of functional enrichment analysis indicated notable associations between the signature genes and pathways related to the immune system. However, some limitations of our study need to be clarified. Although the differential expression of the identified genes was validated by qRT‐PCR analysis in our study, more prospective investigations are needed to validate the predictive power. In addition, the underlying molecular mechanisms of the genes were not fully understood, which will be the focus of our future research efforts.

## 5 CONCLUSION

We identified a novel signature associated with CRGs, which showed a strong diagnostic value for MM with AUC values exceeding 0.80 across 10 data sets. Functional enrichment analysis indicated that the signature genes might enhance the survival and progression of myeloma cells by influencing immune‐related signaling pathways. Our study highlights the involvement of CRGs in the diagnosis of MM and investigates the underlying molecular mechanisms, offering novel insights into the pathogenesis and progression of MM.

## AUTHOR CONTRIBUTIONS

**Yidong Zhu**: Conceptualization (lead); writing—original draft (equal); formal analysis (lead). **Shuaikang Chang**: Experimental validation (equal); writing—original draft (equal). **Jun Liu**: Writing—review and editing (equal). **Bo Wang**: Writing—review and editing (equal); experimental validation (equal).

## CONFLICT OF INTEREST STATEMENT

The authors declare no conflict of interest.

## Supporting information

Supporting information.

Supporting information.

Supporting information.

Supporting information.

## Data Availability

The data sets used and/or analyzed during the current study are available from the corresponding author on reasonable request.
