# Supplementary material for: Identification of a novel cuproptosis‐related gene signature for multiple myeloma diagnosis
Source: Immun Inflamm Dis. 2023 Nov 7;11(11):e1058. doi: 10.1002/iid3.1058 (PMC10629272; doi:10.1002/iid3.1058)
Supplement: Supplementary file 1 — Supporting information. [file IID3-11-e1058-s003.doc]

**Supplementary Table S1.** Characteristics of the studied datasets.

| **GEO series** | **Control samples** | **MM samples** | **Total samples** |
| --- | --- | --- | --- |
| GSE5900 | 22 | 56 | 78 |
| GSE6477 | 15 | 147 | 162 |
| GSE24870 | 20 | 23 | 43 |
| GSE27838 | 16 | 16 | 32 |
| GSE46053 | 16 | 21 | 37 |
| GSE113295 | 6 | 12 | 18 |
| GSE113736 | 12 | 12 | 24 |
| GSE118985 | 68 | 682 | 750 |
| GSE133346 | 12 | 12 | 24 |
| GSE146649 | 10 | 31 | 41 |
