# Supplementary material for: Identification of a novel cuproptosis‐related gene signature for multiple myeloma diagnosis
Source: Immun Inflamm Dis. 2023 Nov 7;11(11):e1058. doi: 10.1002/iid3.1058 (PMC10629272; doi:10.1002/iid3.1058)
Supplement: Supplementary file 2 — Supporting information. [file IID3-11-e1058-s004.doc]

**Supplementary Table S2. Cuproptosis-related Genes**

| **Cuproptosis-related Genes** |
| --- |
| NFE2L2 |
| NLRP3 |
| ATP7B |
| ATP7A |
| SLC31A1 |
| FDX1 |
| LIAS |
| LIPT1 |
| LIPT2 |
| DLD |
| DLAT |
| PDHA1 |
| PDHB |
| MTF1 |
| GLS |
| CDKN2A |
| DBT |
| GCSH |
| DLST |
