# Supplementary material for: Identification of a novel cuproptosis‐related gene signature for multiple myeloma diagnosis
Source: Immun Inflamm Dis. 2023 Nov 7;11(11):e1058. doi: 10.1002/iid3.1058 (PMC10629272; doi:10.1002/iid3.1058)
Supplement: Supplementary file 3 — Supporting information. [file IID3-11-e1058-s001.doc]

**Supplementary Table S3.** Detailed information of the differentially expressed cuproptosis-related genes.

| **Gene** | **conMean** | **treatMean** | **logFC** | **pvalue** | **fdr** |
| --- | --- | --- | --- | --- | --- |
| NLRP3 | 5.935612 | 5.272823 | -0.66279 | 1.51E-12 | 5.12E-12 |
| ATP7B | 9.425311 | 9.06454 | -0.36077 | 1.37E-11 | 3.88E-11 |
| ATP7A | 9.398463 | 9.220756 | -0.17771 | 0.001256 | 0.001942 |
| FDX1 | 8.758205 | 9.11669 | 0.358486 | 1.03E-09 | 2.18E-09 |
| LIPT1 | 9.453987 | 9.550046 | 0.096059 | 0.031803 | 0.045054 |
| DLD | 10.35322 | 10.01826 | -0.33497 | 1.88E-11 | 4.57E-11 |
| PDHA1 | 11.16417 | 10.84663 | -0.31754 | 9.00E-17 | 1.53E-15 |
| PDHB | 10.47869 | 10.53269 | 0.054001 | 8.08E-05 | 0.000137 |
| MTF1 | 9.994085 | 9.699806 | -0.29428 | 5.49E-13 | 2.33E-12 |
| GLS | 8.115303 | 8.408482 | 0.293179 | 1.12E-08 | 2.11E-08 |
| CDKN2A | 6.256651 | 7.022429 | 0.765778 | 1.10E-15 | 9.32E-15 |
| DLST | 10.22855 | 9.8205 | -0.40805 | 4.76E-13 | 2.33E-12 |
