# Supplementary material for: Identification of a novel cuproptosis‐related gene signature for multiple myeloma diagnosis
Source: Immun Inflamm Dis. 2023 Nov 7;11(11):e1058. doi: 10.1002/iid3.1058 (PMC10629272; doi:10.1002/iid3.1058)
Supplement: Supplementary file 4 — Supporting information. [file IID3-11-e1058-s002.doc]

**Supplementary Table S4**. Specific details of the ceRNA network.

| **miRNA** | **Gene** |  | **Type** |
| --- | --- | --- | --- |
| hsa-miR-195-3p | MTF1 |  | mRNA |
| hsa-miR-335-5p | PDHA1 |  | mRNA |
| hsa-miR-542-3p | PDHA1 |  | mRNA |
| hsa-miR-3145-3p | ATP7A |  | mRNA |
| hsa-miR-527 | PDHA1 |  | mRNA |
| hsa-miR-4282 | FDX1 |  | mRNA |
| hsa-miR-4293 | MTF1 |  | mRNA |
| hsa-miR-101-5p | FDX1 |  | mRNA |
| hsa-miR-335-3p | ATP7A |  | mRNA |
| hsa-miR-485-5p | MTF1 |  | mRNA |
| hsa-miR-589-5p | PDHA1 |  | mRNA |
| hsa-miR-148a-5p | DLST |  | mRNA |
| hsa-miR-1257 | CDKN2A |  | mRNA |
| hsa-miR-4282 | ATP7A |  | mRNA |
| hsa-miR-367-3p | MTF1 |  | mRNA |
| hsa-miR-1305 | ATP7A |  | mRNA |
| hsa-miR-129-5p | MTF1 |  | mRNA |
| hsa-miR-3176 | MTF1 |  | mRNA |
| hsa-miR-4275 | PDHA1 |  | mRNA |
| hsa-miR-302b-3p | MTF1 |  | mRNA |
| hsa-miR-661 | MTF1 |  | mRNA |
| hsa-miR-647 | MTF1 |  | mRNA |
| hsa-miR-663a | CDKN2A |  | mRNA |
| hsa-miR-93-5p | MTF1 |  | mRNA |
| hsa-miR-340-5p | ATP7A |  | mRNA |
| hsa-miR-421 | MTF1 |  | mRNA |
| hsa-miR-223-3p | ATP7A |  | mRNA |
| hsa-miR-455-5p | ATP7A |  | mRNA |
| hsa-miR-106b-5p | MTF1 |  | mRNA |
| hsa-miR-3148 | MTF1 |  | mRNA |
| hsa-miR-548m | PDHA1 |  | mRNA |
| hsa-miR-506-3p | MTF1 |  | mRNA |
| hsa-miR-3133 | MTF1 |  | mRNA |
| hsa-let-7a-5p | DLST |  | mRNA |
| hsa-miR-374b-3p | ATP7A |  | mRNA |
| hsa-miR-4327 | MTF1 |  | mRNA |
| hsa-miR-1299 | PDHA1 |  | mRNA |
| hsa-miR-373-5p | PDHA1 |  | mRNA |
| hsa-miR-1276 | PDHA1 |  | mRNA |
| hsa-miR-30b-3p | MTF1 |  | mRNA |
| hsa-miR-374b-3p | MTF1 |  | mRNA |
| hsa-miR-92b-3p | ATP7A |  | mRNA |
| hsa-miR-548h-5p | MTF1 |  | mRNA |
| hsa-let-7a-3p | PDHB |  | mRNA |
| hsa-miR-548c-5p | MTF1 |  | mRNA |
| hsa-miR-223-5p | ATP7A |  | mRNA |
| hsa-miR-761 | MTF1 |  | mRNA |
| hsa-miR-765 | PDHB |  | mRNA |
| hsa-miR-424-5p | ATP7A |  | mRNA |
| hsa-miR-513a-5p | MTF1 |  | mRNA |
| hsa-miR-3202 | MTF1 |  | mRNA |
| hsa-miR-24-3p | MTF1 |  | mRNA |
| hsa-miR-548k | MTF1 |  | mRNA |
| hsa-miR-4269 | MTF1 |  | mRNA |
| hsa-miR-877-3p | ATP7A |  | mRNA |
| hsa-miR-590-3p | FDX1 |  | mRNA |
| hsa-miR-1283 | ATP7A |  | mRNA |
| hsa-miR-335-3p | PDHA1 |  | mRNA |
| hsa-miR-1227-3p | ATP7A |  | mRNA |
| hsa-miR-18a-3p | DLST |  | mRNA |
| hsa-miR-607 | MTF1 |  | mRNA |
| hsa-miR-606 | FDX1 |  | mRNA |
| hsa-miR-548d-3p | ATP7A |  | mRNA |
| hsa-let-7b-3p | MTF1 |  | mRNA |
| hsa-miR-649 | MTF1 |  | mRNA |
| hsa-miR-4275 | ATP7A |  | mRNA |
| hsa-miR-944 | FDX1 |  | mRNA |
| hsa-miR-33a-3p | PDHA1 |  | mRNA |
| hsa-miR-346 | DLST |  | mRNA |
| hsa-miR-584-5p | PDHA1 |  | mRNA |
| hsa-miR-539-5p | MTF1 |  | mRNA |
| hsa-let-7i-5p | DLST |  | mRNA |
| hsa-miR-338-5p | ATP7A |  | mRNA |
| hsa-miR-548b-5p | MTF1 |  | mRNA |
| hsa-miR-634 | DLST |  | mRNA |
| hsa-miR-1228-3p | DLST |  | mRNA |
| hsa-miR-139-5p | ATP7A |  | mRNA |
| hsa-miR-148b-3p | ATP7A |  | mRNA |
| hsa-miR-9-5p | ATP7A |  | mRNA |
| hsa-miR-4307 | ATP7A |  | mRNA |
| hsa-miR-495-3p | PDHB |  | mRNA |
| hsa-miR-570-3p | ATP7A |  | mRNA |
| hsa-miR-922 | MTF1 |  | mRNA |
| hsa-miR-302d-5p | DLST |  | mRNA |
| hsa-miR-548w | MTF1 |  | mRNA |
| hsa-miR-25-3p | ATP7A |  | mRNA |
| hsa-miR-518a-5p | PDHA1 |  | mRNA |
| hsa-miR-542-5p | CDKN2A |  | mRNA |
| hsa-let-7f-2-3p | ATP7A |  | mRNA |
| hsa-let-7g-5p | DLST |  | mRNA |
| hsa-miR-515-5p | MTF1 |  | mRNA |
| hsa-miR-214-3p | MTF1 |  | mRNA |
| hsa-miR-2278 | PDHA1 |  | mRNA |
| hsa-miR-520d-3p | MTF1 |  | mRNA |
| hsa-miR-559 | MTF1 |  | mRNA |
| hsa-miR-876-5p | MTF1 |  | mRNA |
| hsa-miR-491-3p | MTF1 |  | mRNA |
| hsa-miR-663b | CDKN2A |  | mRNA |
| hsa-let-7b-5p | DLST |  | mRNA |
| hsa-miR-495-3p | PDHA1 |  | mRNA |
| hsa-miR-568 | PDHA1 |  | mRNA |
| hsa-miR-92b-3p | MTF1 |  | mRNA |
| hsa-miR-548t-5p | MTF1 |  | mRNA |
| hsa-miR-502-5p | DLST |  | mRNA |
| hsa-miR-142-5p | ATP7A |  | mRNA |
| hsa-miR-3125 | MTF1 |  | mRNA |
| hsa-miR-15b-3p | PDHA1 |  | mRNA |
| hsa-miR-1179 | FDX1 |  | mRNA |
| hsa-miR-1185-5p | PDHB |  | mRNA |
| hsa-let-7d-5p | DLST |  | mRNA |
| hsa-miR-192-3p | ATP7A |  | mRNA |
| hsa-miR-4328 | DLST |  | mRNA |
| hsa-miR-302e | MTF1 |  | mRNA |
| hsa-miR-519e-5p | MTF1 |  | mRNA |
| hsa-miR-206 | PDHA1 |  | mRNA |
| hsa-miR-513a-3p | PDHB |  | mRNA |
| hsa-miR-3133 | PDHB |  | mRNA |
| hsa-miR-200a-5p | ATP7A |  | mRNA |
| hsa-miR-340-5p | FDX1 |  | mRNA |
| hsa-miR-15b-5p | ATP7A |  | mRNA |
| hsa-miR-1323 | PDHB |  | mRNA |
| hsa-miR-23b-3p | MTF1 |  | mRNA |
| hsa-let-7a-3p | MTF1 |  | mRNA |
| hsa-miR-127-5p | ATP7A |  | mRNA |
| hsa-miR-603 | PDHA1 |  | mRNA |
| hsa-miR-607 | ATP7A |  | mRNA |
| hsa-miR-30d-3p | DLST |  | mRNA |
| hsa-miR-520a-3p | MTF1 |  | mRNA |
| hsa-miR-509-5p | ATP7A |  | mRNA |
| hsa-miR-633 | PDHA1 |  | mRNA |
| hsa-miR-3163 | ATP7A |  | mRNA |
| hsa-miR-569 | PDHA1 |  | mRNA |
| hsa-miR-590-5p | FDX1 |  | mRNA |
| hsa-miR-590-3p | ATP7A |  | mRNA |
| hsa-miR-939-5p | MTF1 |  | mRNA |
| hsa-miR-10a-5p | MTF1 |  | mRNA |
| hsa-miR-194-5p | PDHB |  | mRNA |
| hsa-miR-297 | ATP7A |  | mRNA |
| hsa-miR-148b-3p | MTF1 |  | mRNA |
| hsa-miR-1976 | PDHA1 |  | mRNA |
| hsa-miR-27b-5p | PDHA1 |  | mRNA |
| hsa-miR-4310 | MTF1 |  | mRNA |
| hsa-miR-888-5p | ATP7A |  | mRNA |
| hsa-miR-205-5p | ATP7A |  | mRNA |
| hsa-miR-338-5p | MTF1 |  | mRNA |
| hsa-miR-28-5p | MTF1 |  | mRNA |
| hsa-miR-557 | MTF1 |  | mRNA |
| hsa-miR-192-3p | PDHA1 |  | mRNA |
| hsa-miR-3123 | FDX1 |  | mRNA |
| hsa-miR-622 | FDX1 |  | mRNA |
| hsa-let-7b-3p | PDHB |  | mRNA |
| hsa-miR-125b-5p | MTF1 |  | mRNA |
| hsa-miR-506-3p | ATP7A |  | mRNA |
| hsa-miR-149-3p | FDX1 |  | mRNA |
| hsa-miR-448 | MTF1 |  | mRNA |
| hsa-miR-607 | PDHB |  | mRNA |
| hsa-miR-495-3p | ATP7A |  | mRNA |
| hsa-miR-938 | DLST |  | mRNA |
| hsa-miR-548c-3p | ATP7A |  | mRNA |
| hsa-miR-4307 | PDHA1 |  | mRNA |
| hsa-miR-1237-3p | ATP7A |  | mRNA |
| hsa-miR-32-5p | ATP7A |  | mRNA |
| hsa-miR-3156-5p | PDHA1 |  | mRNA |
| hsa-miR-21-5p | PDHA1 |  | mRNA |
| hsa-let-7f-2-3p | PDHB |  | mRNA |
| hsa-miR-1256 | FDX1 |  | mRNA |
| hsa-miR-18a-3p | MTF1 |  | mRNA |
| hsa-miR-548p | MTF1 |  | mRNA |
| hsa-miR-145-5p | MTF1 |  | mRNA |
| hsa-miR-4310 | FDX1 |  | mRNA |
| hsa-miR-16-2-3p | MTF1 |  | mRNA |
| hsa-miR-3158-3p | ATP7A |  | mRNA |
| hsa-miR-518c-5p | MTF1 |  | mRNA |
| hsa-miR-590-3p | PDHB |  | mRNA |
| hsa-miR-20b-5p | MTF1 |  | mRNA |
| hsa-miR-4263 | ATP7A |  | mRNA |
| hsa-miR-4276 | ATP7A |  | mRNA |
| hsa-miR-26b-3p | ATP7A |  | mRNA |
| hsa-miR-130a-5p | FDX1 |  | mRNA |
| hsa-miR-21-3p | MTF1 |  | mRNA |
| hsa-miR-10b-3p | MTF1 |  | mRNA |
| hsa-miR-4271 | MTF1 |  | mRNA |
| hsa-miR-148a-3p | MTF1 |  | mRNA |
| hsa-miR-302b-5p | DLST |  | mRNA |
| hsa-let-7f-5p | DLST |  | mRNA |
| hsa-miR-613 | PDHA1 |  | mRNA |
| hsa-miR-1205 | DLST |  | mRNA |
| hsa-miR-373-3p | MTF1 |  | mRNA |
| hsa-miR-1264 | PDHA1 |  | mRNA |
| hsa-miR-516a-3p | DLST |  | mRNA |
| hsa-miR-576-5p | ATP7A |  | mRNA |
| hsa-miR-125a-5p | MTF1 |  | mRNA |
| hsa-miR-186-5p | MTF1 |  | mRNA |
| hsa-miR-1275 | ATP7A |  | mRNA |
| hsa-miR-570-3p | FDX1 |  | mRNA |
| hsa-miR-4256 | MTF1 |  | mRNA |
| hsa-miR-140-5p | DLST |  | mRNA |
| hsa-miR-149-3p | MTF1 |  | mRNA |
| hsa-miR-520c-3p | MTF1 |  | mRNA |
| hsa-miR-4269 | DLST |  | mRNA |
| hsa-miR-4284 | FDX1 |  | mRNA |
| hsa-miR-770-5p | MTF1 |  | mRNA |
| hsa-let-7f-1-3p | PDHB |  | mRNA |
| hsa-miR-708-5p | MTF1 |  | mRNA |
| hsa-miR-875-3p | MTF1 |  | mRNA |
| hsa-miR-98-5p | DLST |  | mRNA |
| hsa-miR-944 | ATP7A |  | mRNA |
| hsa-miR-4303 | MTF1 |  | mRNA |
| hsa-miR-1-3p | PDHA1 |  | mRNA |
| hsa-miR-802 | MTF1 |  | mRNA |
| hsa-miR-4302 | CDKN2A |  | mRNA |
| hsa-miR-409-3p | ATP7A |  | mRNA |
| hsa-miR-940 | DLST |  | mRNA |
| hsa-miR-548i | MTF1 |  | mRNA |
| hsa-miR-548l | MTF1 |  | mRNA |
| hsa-let-7e-5p | DLST |  | mRNA |
| hsa-miR-21-5p | FDX1 |  | mRNA |
| hsa-miR-545-3p | MTF1 |  | mRNA |
| hsa-miR-101-5p | PDHB |  | mRNA |
| hsa-miR-4291 | MTF1 |  | mRNA |
| hsa-miR-4270 | DLST |  | mRNA |
| hsa-miR-187-5p | ATP7A |  | mRNA |
| hsa-miR-1207-5p | ATP7A |  | mRNA |
| hsa-miR-924 | DLST |  | mRNA |
| hsa-miR-497-5p | ATP7A |  | mRNA |
| hsa-miR-507 | MTF1 |  | mRNA |
| hsa-miR-488-3p | MTF1 |  | mRNA |
| hsa-miR-596 | ATP7A |  | mRNA |
| hsa-miR-764 | DLST |  | mRNA |
| hsa-miR-4281 | DLST |  | mRNA |
| hsa-miR-3201 | ATP7A |  | mRNA |
| hsa-miR-129-5p | ATP7A |  | mRNA |
| hsa-miR-640 | ATP7A |  | mRNA |
| hsa-miR-1284 | ATP7A |  | mRNA |
| hsa-miR-15a-5p | ATP7A |  | mRNA |
| hsa-miR-302b-5p | ATP7A |  | mRNA |
| hsa-miR-10b-5p | MTF1 |  | mRNA |
| hsa-miR-641 | FDX1 |  | mRNA |
| hsa-miR-367-3p | ATP7A |  | mRNA |
| hsa-miR-369-3p | FDX1 |  | mRNA |
| hsa-miR-197-3p | DLST |  | mRNA |
| hsa-miR-127-5p | FDX1 |  | mRNA |
| hsa-miR-505-5p | MTF1 |  | mRNA |
| hsa-miR-373-5p | MTF1 |  | mRNA |
| hsa-miR-302d-3p | MTF1 |  | mRNA |
| hsa-miR-23a-3p | MTF1 |  | mRNA |
| hsa-miR-365a-3p | MTF1 |  | mRNA |
| hsa-miR-149-3p | ATP7A |  | mRNA |
| hsa-miR-3134 | MTF1 |  | mRNA |
| hsa-miR-2114-3p | PDHA1 |  | mRNA |
| hsa-miR-3190-5p | MTF1 |  | mRNA |
| hsa-miR-1207-5p | MTF1 |  | mRNA |
| hsa-miR-3163 | FDX1 |  | mRNA |
| hsa-miR-9-3p | PDHA1 |  | mRNA |
| hsa-miR-4273 | FDX1 |  | mRNA |
| hsa-miR-4270 | ATP7A |  | mRNA |
| hsa-miR-125b-1-3p | MTF1 |  | mRNA |
| hsa-miR-3140-3p | MTF1 |  | mRNA |
| hsa-miR-1291 | CDKN2A |  | mRNA |
| hsa-miR-25-3p | MTF1 |  | mRNA |
| hsa-miR-149-5p | MTF1 |  | mRNA |
| hsa-miR-449c-3p | MTF1 |  | mRNA |
| hsa-miR-34b-3p | FDX1 |  | mRNA |
| hsa-miR-488-3p | ATP7A |  | mRNA |
| hsa-miR-222-5p | FDX1 |  | mRNA |
| hsa-miR-4312 | PDHA1 |  | mRNA |
| hsa-miR-3130-5p | FDX1 |  | mRNA |
| hsa-miR-3123 | PDHA1 |  | mRNA |
| hsa-miR-195-3p | ATP7A |  | mRNA |
| hsa-miR-2054 | FDX1 |  | mRNA |
| hsa-miR-4288 | DLST |  | mRNA |
| hsa-let-7f-1-3p | MTF1 |  | mRNA |
| hsa-miR-2115-3p | MTF1 |  | mRNA |
| hsa-miR-548a-5p | MTF1 |  | mRNA |
| hsa-miR-363-3p | MTF1 |  | mRNA |
| hsa-miR-1272 | DLST |  | mRNA |
| hsa-miR-449c-5p | MTF1 |  | mRNA |
| hsa-miR-486-3p | PDHA1 |  | mRNA |
| hsa-miR-576-5p | FDX1 |  | mRNA |
| hsa-miR-9-3p | FDX1 |  | mRNA |
| hsa-miR-1248 | ATP7A |  | mRNA |
| hsa-miR-1286 | CDKN2A |  | mRNA |
| hsa-miR-195-5p | ATP7A |  | mRNA |
| hsa-miR-148a-3p | ATP7A |  | mRNA |
| hsa-miR-650 | PDHA1 |  | mRNA |
| hsa-miR-577 | MTF1 |  | mRNA |
| hsa-miR-362-3p | DLST |  | mRNA |
| hsa-miR-4325 | MTF1 |  | mRNA |
| hsa-miR-148b-5p | DLST |  | mRNA |
| hsa-miR-302d-5p | ATP7A |  | mRNA |
| hsa-miR-3065-3p | DLST |  | mRNA |
| hsa-miR-205-3p | PDHB |  | mRNA |
| hsa-miR-16-5p | ATP7A |  | mRNA |
| hsa-miR-495-3p | FDX1 |  | mRNA |
| hsa-miR-32-5p | MTF1 |  | mRNA |
| hsa-miR-548a-3p | MTF1 |  | mRNA |
| hsa-miR-302a-3p | MTF1 |  | mRNA |
| hsa-miR-16-2-3p | ATP7A |  | mRNA |
| hsa-miR-1278 | MTF1 |  | mRNA |
| hsa-miR-509-3-5p | ATP7A |  | mRNA |
| hsa-miR-10b-3p | PDHA1 |  | mRNA |
| hsa-miR-30b-3p | FDX1 |  | mRNA |
| hsa-miR-106a-5p | MTF1 |  | mRNA |
| hsa-miR-548d-5p | MTF1 |  | mRNA |
| hsa-miR-22-3p | MTF1 |  | mRNA |
| hsa-miR-9-3p | ATP7A |  | mRNA |
| hsa-miR-1237-3p | MTF1 |  | mRNA |
| hsa-miR-3190-3p | MTF1 |  | mRNA |
| hsa-miR-363-3p | ATP7A |  | mRNA |
| hsa-miR-135b-3p | ATP7A |  | mRNA |
| hsa-miR-4251 | MTF1 |  | mRNA |
| hsa-miR-1258 | ATP7A |  | mRNA |
| hsa-miR-1248 | MTF1 |  | mRNA |
| hsa-miR-802 | CTA-414D7.1 |  | lncRNA |
| hsa-miR-194-5p | CTA-414D7.1 |  | lncRNA |
| hsa-miR-765 | GAS6-AS1 |  | lncRNA |
| hsa-miR-570-3p | RP11-10J21.4 |  | lncRNA |
| hsa-miR-875-3p | CDR1-AS |  | lncRNA |
| hsa-miR-214-3p | LA16c-306A4.2 |  | lncRNA |
| hsa-miR-192-3p | RP11-830F9.6 |  | lncRNA |
| hsa-miR-1228-3p | RP11-210M15.1 |  | lncRNA |
| hsa-miR-197-3p | LA16c-306A4.2 |  | lncRNA |
| hsa-miR-515-5p | TTLL10-AS1 |  | lncRNA |
| hsa-miR-1228-3p | CITF22-1A6.3 |  | lncRNA |
| hsa-miR-30b-3p | C10orf91 |  | lncRNA |
| hsa-miR-149-3p | C10orf91 |  | lncRNA |
| hsa-miR-145-5p | MUC19 |  | lncRNA |
| hsa-miR-421 | AC079779.7 |  | lncRNA |
| hsa-miR-223-5p | AC069257.8 |  | lncRNA |
| hsa-miR-449c-5p | AC079779.7 |  | lncRNA |
| hsa-miR-26b-3p | LA16c-OS12.2 |  | lncRNA |
| hsa-miR-149-5p | LINC01043 |  | lncRNA |
| hsa-miR-1227-3p | HP09025 |  | lncRNA |
| hsa-miR-1-3p | LINC01043 |  | lncRNA |
| hsa-miR-515-5p | AC079779.7 |  | lncRNA |
| hsa-miR-876-5p | CDR1-AS |  | lncRNA |
| hsa-miR-148a-3p | CITF22-1A6.3 |  | lncRNA |
| hsa-miR-24-3p | RP11-102K13.5 |  | lncRNA |
| hsa-miR-545-3p | LINC01070 |  | lncRNA |
| hsa-miR-365a-3p | LINC01070 |  | lncRNA |
| hsa-miR-449c-5p | RP11-138B4.1 |  | lncRNA |
| hsa-miR-486-3p | C10orf91 |  | lncRNA |
| hsa-miR-1227-3p | RP11-830F9.6 |  | lncRNA |
| hsa-miR-765 | RP11-138B4.1 |  | lncRNA |
| hsa-let-7a-3p | RP3-323A16.1 |  | lncRNA |
| hsa-miR-939-5p | AATBC |  | lncRNA |
| hsa-miR-186-5p | RP11-99L13.2 |  | lncRNA |
| hsa-miR-650 | RP11-138B4.1 |  | lncRNA |
| hsa-miR-223-5p | RP3-323A16.1 |  | lncRNA |
| hsa-miR-1237-3p | GS1-519E5.1 |  | lncRNA |
| hsa-miR-22-3p | RP11-326C3.10 |  | lncRNA |
| hsa-miR-197-3p | LINC01070 |  | lncRNA |
| hsa-miR-24-3p | LINC01106 |  | lncRNA |
| hsa-miR-365a-3p | HP09025 |  | lncRNA |
| hsa-miR-18a-3p | RP3-388N13.3 |  | lncRNA |
| hsa-miR-125b-1-3p | TP73-AS1 |  | lncRNA |
| hsa-miR-1228-3p | RP4-737E23.2 |  | lncRNA |
| hsa-let-7a-3p | DPP10-AS2 |  | lncRNA |
| hsa-miR-650 | RP5-892K4.1 |  | lncRNA |
| hsa-miR-10a-5p | RP5-892K4.1 |  | lncRNA |
| hsa-miR-576-5p | CTC-459F4.1 |  | lncRNA |
| hsa-miR-214-3p | RP4-539M6.22 |  | lncRNA |
| hsa-miR-15b-3p | AC011284.3 |  | lncRNA |
| hsa-miR-640 | AC069257.8 |  | lncRNA |
| hsa-miR-449c-5p | RP11-458F8.4 |  | lncRNA |
| hsa-miR-939-5p | RP11-627G23.1 |  | lncRNA |
| hsa-miR-765 | RP11-627G23.1 |  | lncRNA |
| hsa-miR-515-5p | RP13-507P19.2 |  | lncRNA |
| hsa-miR-148a-3p | LA16c-306A4.2 |  | lncRNA |
| hsa-miR-140-5p | MUC2 |  | lncRNA |
| hsa-miR-186-5p | MIR325HG |  | lncRNA |
| hsa-miR-650 | AIRN |  | lncRNA |
| hsa-miR-488-3p | RP5-892K4.1 |  | lncRNA |
| hsa-miR-28-5p | XXyac-YM21GA2.7 |  | lncRNA |
| hsa-miR-1207-5p | C10orf91 |  | lncRNA |
| hsa-miR-28-5p | CTD-2245F17.9 |  | lncRNA |
| hsa-miR-130a-5p | LL22NC03-27C5.1 |  | lncRNA |
| hsa-miR-1207-5p | RP11-618K13.2 |  | lncRNA |
| hsa-miR-18a-3p | GAS8-AS1 |  | lncRNA |
| hsa-miR-1976 | RP13-580B18.4 |  | lncRNA |
| hsa-miR-129-5p | RP11-166B2.5 |  | lncRNA |
| hsa-miR-877-3p | LINC00689 |  | lncRNA |
| hsa-miR-622 | RP13-507P19.2 |  | lncRNA |
| hsa-miR-22-3p | RP11-326C3.14 |  | lncRNA |
| hsa-miR-149-3p | CTA-941F9.10 |  | lncRNA |
| hsa-miR-542-5p | RP13-507P19.2 |  | lncRNA |
| hsa-miR-149-5p | RP11-394A14.2 |  | lncRNA |
| hsa-miR-30b-3p | MUC2 |  | lncRNA |
| hsa-miR-1207-5p | LINC00265 |  | lncRNA |
| hsa-miR-1207-5p | RP11-333E1.2 |  | lncRNA |
| hsa-miR-28-5p | CTD-2619J13.14 |  | lncRNA |
| hsa-miR-149-3p | AIRN |  | lncRNA |
| hsa-miR-622 | CTC-459F4.1 |  | lncRNA |
| hsa-let-7a-3p | FAM230B |  | lncRNA |
| hsa-miR-505-5p | CTB-51J22.1 |  | lncRNA |
| hsa-miR-335-3p | SLC8A1-AS1 |  | lncRNA |
| hsa-miR-28-5p | RP5-991B18.1 |  | lncRNA |
| hsa-miR-875-3p | PCBP3-OT1 |  | lncRNA |
| hsa-miR-939-5p | HP09025 |  | lncRNA |
| hsa-miR-1976 | AC010761.6 |  | lncRNA |
| hsa-miR-302a-3p | RP4-539M6.22 |  | lncRNA |
| hsa-let-7a-5p | RP11-94C24.13 |  | lncRNA |
| hsa-miR-486-3p | RP11-94C24.13 |  | lncRNA |
| hsa-miR-1207-5p | AP001476.4 |  | lncRNA |
| hsa-miR-1207-5p | RP4-539M6.22 |  | lncRNA |
| hsa-miR-148a-3p | RP11-717I24.1 |  | lncRNA |
| hsa-miR-650 | RP11-458F8.4 |  | lncRNA |
| hsa-miR-18a-3p | CTD-2619J13.14 |  | lncRNA |
| hsa-miR-515-5p | RP13-580B18.4 |  | lncRNA |
| hsa-miR-223-3p | FAM95B1 |  | lncRNA |
| hsa-miR-542-3p | RP4-671O14.7 |  | lncRNA |
| hsa-miR-421 | LINC01165 |  | lncRNA |
| hsa-miR-28-5p | RP3-388N13.3 |  | lncRNA |
| hsa-miR-542-3p | AC079586.1 |  | lncRNA |
| hsa-miR-545-3p | LINC01165 |  | lncRNA |
| hsa-miR-1228-3p | CTD-3138B18.5 |  | lncRNA |
| hsa-miR-1-3p | RP3-470B24.5 |  | lncRNA |
| hsa-miR-10a-5p | RP11-54O7.17 |  | lncRNA |
| hsa-miR-663b | RP11-54O7.17 |  | lncRNA |
| hsa-miR-149-3p | LINC00265 |  | lncRNA |
| hsa-miR-1976 | LINC01123 |  | lncRNA |
| hsa-miR-28-5p | RP11-458F8.4 |  | lncRNA |
| hsa-miR-149-3p | RP11-311F12.1 |  | lncRNA |
| hsa-miR-939-5p | LINC00173 |  | lncRNA |
| hsa-miR-18a-3p | NNT-AS1 |  | lncRNA |
| hsa-miR-223-5p | LINC00689 |  | lncRNA |
| hsa-miR-542-3p | RP11-157B13.7 |  | lncRNA |
| hsa-miR-149-3p | LINC00689 |  | lncRNA |
| hsa-miR-650 | LINC00689 |  | lncRNA |
| hsa-miR-129-5p | AC006548.28 |  | lncRNA |
| hsa-miR-650 | LINC00265 |  | lncRNA |
| hsa-miR-877-3p | LINC00940 |  | lncRNA |
| hsa-miR-145-5p | CTD-3099C6.5 |  | lncRNA |
| hsa-miR-770-5p | CTD-3099C6.5 |  | lncRNA |
| hsa-miR-140-5p | MUC19 |  | lncRNA |
| hsa-miR-148a-3p | RP1-182D15.2 |  | lncRNA |
| hsa-miR-1976 | LINC01523 |  | lncRNA |
| hsa-miR-515-5p | SPACA6P |  | lncRNA |
| hsa-miR-223-5p | RP11-426C22.4 |  | lncRNA |
| hsa-miR-515-5p | AC093642.4 |  | lncRNA |
| hsa-miR-148a-3p | SNHG14 |  | lncRNA |
| hsa-miR-1976 | CTD-3032J10.2 |  | lncRNA |
| hsa-miR-186-5p | LINC00613 |  | lncRNA |
| hsa-miR-650 | RP11-304L19.13 |  | lncRNA |
| hsa-miR-18a-3p | CTD-2517M22.17 |  | lncRNA |
| hsa-miR-924 | LINC00632 |  | lncRNA |
| hsa-miR-223-5p | HPVC1 |  | lncRNA |
| hsa-miR-106a-5p | LINC01106 |  | lncRNA |
| hsa-miR-650 | RP5-1014D13.2 |  | lncRNA |
| hsa-miR-297 | CTA-929C8.6 |  | lncRNA |
| hsa-miR-542-3p | LINC00917 |  | lncRNA |
| hsa-miR-106a-5p | RP11-369C8.1 |  | lncRNA |
| hsa-miR-539-5p | AC018816.3 |  | lncRNA |
| hsa-miR-1207-5p | RP11-680F20.6 |  | lncRNA |
| hsa-miR-30b-3p | LINCMD1 |  | lncRNA |
| hsa-miR-186-5p | DYX1C1-CCPG1 |  | lncRNA |
| hsa-miR-640 | MUC19 |  | lncRNA |
| hsa-miR-30b-3p | RP11-153F5.7 |  | lncRNA |
| hsa-miR-515-5p | FAM74A1 |  | lncRNA |
| hsa-miR-1976 | TMEM191C |  | lncRNA |
| hsa-miR-1237-3p | AC091153.4 |  | lncRNA |
| hsa-miR-505-5p | AC016682.1 |  | lncRNA |
| hsa-miR-214-3p | TTLL10-AS1 |  | lncRNA |
| hsa-miR-875-3p | RP11-64K12.8 |  | lncRNA |
| hsa-miR-214-3p | AC015849.16 |  | lncRNA |
| hsa-miR-223-5p | RP11-243A14.1 |  | lncRNA |
| hsa-miR-1976 | LINC00174 |  | lncRNA |
| hsa-miR-1207-5p | RP11-867G23.4 |  | lncRNA |
| hsa-miR-18a-3p | ADGRA1-AS1 |  | lncRNA |
| hsa-miR-650 | RP11-66B24.2 |  | lncRNA |
| hsa-miR-875-3p | FRMPD3-AS1 |  | lncRNA |
| hsa-miR-30b-3p | AC011284.3 |  | lncRNA |
| hsa-miR-214-3p | TMEM9B-AS1 |  | lncRNA |
| hsa-miR-186-5p | RP11-154D6.1 |  | lncRNA |
| hsa-miR-1237-3p | RP11-90K6.1 |  | lncRNA |
| hsa-miR-765 | ST20-AS1 |  | lncRNA |
| hsa-miR-650 | CTD-2013N17.7 |  | lncRNA |
| hsa-miR-939-5p | VPS9D1-AS1 |  | lncRNA |
| hsa-miR-1976 | RP11-627G23.1 |  | lncRNA |
| hsa-miR-130a-5p | RP11-210M15.1 |  | lncRNA |
| hsa-miR-149-3p | CTA-315H11.2 |  | lncRNA |
| hsa-miR-18a-3p | RP5-1029F21.2 |  | lncRNA |
| hsa-miR-149-3p | RP11-153F5.7 |  | lncRNA |
| hsa-miR-149-3p | LINC00173 |  | lncRNA |
| hsa-miR-939-5p | CTA-941F9.10 |  | lncRNA |
| hsa-miR-139-5p | AC015849.16 |  | lncRNA |
| hsa-miR-186-5p | RP11-22A3.2 |  | lncRNA |
| hsa-miR-939-5p | RP11-311F12.1 |  | lncRNA |
| hsa-miR-26b-3p | RP13-143G15.4 |  | lncRNA |
| hsa-miR-590-3p | LINC00240 |  | lncRNA |
| hsa-miR-186-5p | SFTPD-AS1 |  | lncRNA |
| hsa-miR-18a-3p | RP11-573D15.2 |  | lncRNA |
| hsa-miR-130a-5p | AC084219.4 |  | lncRNA |
| hsa-miR-18a-3p | RP11-469N6.1 |  | lncRNA |
| hsa-miR-149-3p | TMEM191A |  | lncRNA |
| hsa-miR-1207-5p | LINC00969 |  | lncRNA |
| hsa-miR-650 | RP5-1039K5.19 |  | lncRNA |
| hsa-miR-1976 | LINC01054 |  | lncRNA |
| hsa-miR-18a-3p | AP001062.7 |  | lncRNA |
| hsa-miR-149-3p | RP11-186N15.3 |  | lncRNA |
| hsa-miR-129-5p | LINC00662 |  | lncRNA |
| hsa-miR-515-5p | LINC01002 |  | lncRNA |
| hsa-miR-1207-5p | H19 |  | lncRNA |
| hsa-miR-197-3p | RP11-469N6.1 |  | lncRNA |
| hsa-miR-1207-5p | RP5-1142A6.2 |  | lncRNA |
| hsa-miR-28-5p | NNT-AS1 |  | lncRNA |
| hsa-miR-205-5p | FAR1-IT1 |  | lncRNA |
| hsa-miR-542-3p | LINC01224 |  | lncRNA |
| hsa-miR-939-5p | RP11-278A23.4 |  | lncRNA |
| hsa-miR-335-3p | CTA-392E5.1 |  | lncRNA |
| hsa-miR-28-5p | CTB-51J22.1 |  | lncRNA |
| hsa-miR-590-3p | AC005614.3 |  | lncRNA |
| hsa-miR-127-5p | RP11-325F22.2 |  | lncRNA |
| hsa-miR-924 | SPACA6P |  | lncRNA |
| hsa-miR-197-3p | SNHG14 |  | lncRNA |
| hsa-miR-223-3p | RP1-182D15.2 |  | lncRNA |
| hsa-miR-539-5p | ZNF883 |  | lncRNA |
| hsa-miR-145-5p | CTA-390C10.9 |  | lncRNA |
| hsa-miR-1227-3p | LINC00200 |  | lncRNA |
| hsa-miR-539-5p | CTC-435M10.10 |  | lncRNA |
| hsa-miR-18a-3p | CTD-2245F17.9 |  | lncRNA |
| hsa-miR-1976 | CTB-60B18.18 |  | lncRNA |
| hsa-miR-129-5p | RP11-69I8.2 |  | lncRNA |
| hsa-miR-502-5p | AC010524.2 |  | lncRNA |
| hsa-miR-197-3p | RP1-68D18.2 |  | lncRNA |
| hsa-miR-139-5p | RP11-231D20.2 |  | lncRNA |
| hsa-miR-513a-3p | RP11-474P2.6 |  | lncRNA |
| hsa-miR-125a-5p | MUC19 |  | lncRNA |
| hsa-miR-1228-3p | LINC01043 |  | lncRNA |
| hsa-miR-30b-3p | TTLL10-AS1 |  | lncRNA |
| hsa-miR-650 | RP11-378E13.3 |  | lncRNA |
| hsa-miR-10a-5p | RP11-989E6.10 |  | lncRNA |
| hsa-miR-765 | AC005324.6 |  | lncRNA |
| hsa-miR-939-5p | RP5-1171I10.5 |  | lncRNA |
| hsa-miR-149-3p | CTD-3193O13.1 |  | lncRNA |
| hsa-miR-650 | CTD-2283N19.1 |  | lncRNA |
| hsa-miR-1976 | CTD-2135D7.2 |  | lncRNA |
| hsa-miR-335-3p | RP11-146D12.2 |  | lncRNA |
| hsa-miR-335-3p | RP11-335L23.4 |  | lncRNA |
| hsa-miR-1228-3p | LINC01002 |  | lncRNA |
| hsa-miR-449c-5p | LINC01070 |  | lncRNA |
| hsa-miR-30b-3p | RP11-94C24.13 |  | lncRNA |
| hsa-miR-28-5p | RP4-756G23.5 |  | lncRNA |
| hsa-miR-449c-5p | AC114783.1 |  | lncRNA |
| hsa-miR-590-3p | RP11-762H8.4 |  | lncRNA |
| hsa-miR-515-5p | CTD-2197I11.1 |  | lncRNA |
| hsa-miR-15a-5p | RP11-483P21.6 |  | lncRNA |
| hsa-miR-186-5p | RP1-288H2.2 |  | lncRNA |
| hsa-miR-516a-3p | AC137934.1 |  | lncRNA |
| hsa-miR-515-5p | RP11-1217F2.15 |  | lncRNA |
| hsa-miR-28-5p | RP11-573D15.2 |  | lncRNA |
| hsa-miR-21-3p | RP11-130C6.1 |  | lncRNA |
| hsa-miR-888-5p | AC079799.2 |  | lncRNA |
| hsa-miR-485-5p | AP001626.2 |  | lncRNA |
| hsa-miR-149-3p | MAFG-AS1 |  | lncRNA |
| hsa-miR-214-3p | CTC-242N15.1 |  | lncRNA |
| hsa-miR-145-5p | RP11-717I24.1 |  | lncRNA |
| hsa-miR-130a-5p | AC068489.1 |  | lncRNA |
| hsa-miR-650 | AC074212.5 |  | lncRNA |
| hsa-miR-939-5p | AP001469.9 |  | lncRNA |
| hsa-miR-650 | CTA-243E7.4 |  | lncRNA |
| hsa-miR-876-5p | RP11-699C17.1 |  | lncRNA |
| hsa-miR-129-5p | RP11-67K19.3 |  | lncRNA |
| hsa-miR-449c-5p | LINC00265 |  | lncRNA |
| hsa-miR-1976 | CTD-2126E3.3 |  | lncRNA |
| hsa-miR-765 | ATP2A1-AS1 |  | lncRNA |
| hsa-miR-186-5p | AC124997.1 |  | lncRNA |
| hsa-miR-149-5p | CTD-2008P7.3 |  | lncRNA |
| hsa-miR-1207-5p | LINC01168 |  | lncRNA |
| hsa-miR-129-5p | REV3L-IT1 |  | lncRNA |
| hsa-miR-939-5p | RP11-186N15.3 |  | lncRNA |
| hsa-miR-1237-3p | RP11-407A16.4 |  | lncRNA |
| hsa-miR-1976 | RP11-203B9.4 |  | lncRNA |
| hsa-miR-1-3p | GNG12-AS1 |  | lncRNA |
| hsa-miR-449c-5p | LINC01168 |  | lncRNA |
| hsa-miR-590-3p | AC006548.28 |  | lncRNA |
| hsa-miR-145-5p | AC015849.16 |  | lncRNA |
| hsa-miR-30b-3p | RP11-244B22.11 |  | lncRNA |
| hsa-miR-335-3p | LINC01122 |  | lncRNA |
| hsa-miR-449c-5p | RP11-338K13.1 |  | lncRNA |
| hsa-miR-590-3p | AC093639.1 |  | lncRNA |
| hsa-miR-1207-5p | AC000095.11 |  | lncRNA |
| hsa-miR-485-5p | LINC00265 |  | lncRNA |
| hsa-miR-515-5p | CTD-3099C6.5 |  | lncRNA |
| hsa-miR-214-3p | NR2F1-AS1 |  | lncRNA |
| hsa-miR-149-3p | RP11-148K1.12 |  | lncRNA |
| hsa-miR-650 | EIF3J-AS1 |  | lncRNA |
| hsa-miR-335-5p | SLC8A1-AS1 |  | lncRNA |
| hsa-miR-129-5p | RP5-1125A11.7 |  | lncRNA |
| hsa-miR-340-5p | LINC00869 |  | lncRNA |
| hsa-miR-127-5p | CTB-35F21.1 |  | lncRNA |
| hsa-miR-125a-5p | CTD-3193O13.11 |  | lncRNA |
| hsa-miR-186-5p | CTD-2410N18.4 |  | lncRNA |
| hsa-miR-214-3p | CTC-321K16.1 |  | lncRNA |
| hsa-miR-539-5p | SATB1-AS1 |  | lncRNA |
| hsa-miR-515-5p | SNHG14 |  | lncRNA |
| hsa-miR-149-3p | RP11-430G17.3 |  | lncRNA |
| hsa-miR-129-5p | RP3-508I15.22 |  | lncRNA |
| hsa-miR-18a-3p | UCKL1-AS1 |  | lncRNA |
| hsa-miR-149-3p | RP11-1348G14.8 |  | lncRNA |
| hsa-miR-335-3p | RP11-96K19.4 |  | lncRNA |
| hsa-miR-877-3p | RP1-253P7.1 |  | lncRNA |
| hsa-miR-186-5p | AJ003147.8 |  | lncRNA |
| hsa-miR-130a-5p | LINC00664 |  | lncRNA |
| hsa-miR-539-5p | LINC01539 |  | lncRNA |
| hsa-miR-515-5p | AC084219.4 |  | lncRNA |
| hsa-miR-515-5p | AC015849.16 |  | lncRNA |
| hsa-miR-24-3p | LINC01165 |  | lncRNA |
| hsa-miR-18a-3p | CTD-2523D13.1 |  | lncRNA |
| hsa-miR-449c-5p | RP11-66N24.4 |  | lncRNA |
| hsa-miR-129-5p | SEPSECS-AS1 |  | lncRNA |
| hsa-miR-449c-5p | AP001476.4 |  | lncRNA |
| hsa-miR-9-5p | RP11-397O4.1 |  | lncRNA |
| hsa-miR-186-5p | CTD-3046C4.1 |  | lncRNA |
| hsa-miR-650 | RP3-395M20.8 |  | lncRNA |
| hsa-miR-186-5p | RP11-227H15.4 |  | lncRNA |
| hsa-miR-149-3p | PAX8-AS1 |  | lncRNA |
| hsa-miR-1976 | RP11-304L19.3 |  | lncRNA |
| hsa-miR-944 | RP5-1077H22.2 |  | lncRNA |
| hsa-miR-513a-3p | LL22NC03-N64E9.1 |  | lncRNA |
| hsa-miR-1976 | LINC01001 |  | lncRNA |
| hsa-miR-297 | AC084219.4 |  | lncRNA |
| hsa-let-7a-3p | LPP-AS2 |  | lncRNA |
| hsa-miR-539-5p | RP11-598F7.3 |  | lncRNA |
| hsa-miR-938 | RP11-989E6.10 |  | lncRNA |
| hsa-miR-129-5p | RP11-848P1.3 |  | lncRNA |
| hsa-miR-186-5p | LINC00662 |  | lncRNA |
| hsa-miR-590-3p | CTD-2561J22.5 |  | lncRNA |
| hsa-miR-515-5p | FAM74A7 |  | lncRNA |
| hsa-miR-129-5p | RP11-486O12.2 |  | lncRNA |
| hsa-miR-518a-5p | CTD-2521M24.5 |  | lncRNA |
| hsa-miR-30b-3p | RP11-480I12.10 |  | lncRNA |
| hsa-miR-186-5p | CTB-181F24.1 |  | lncRNA |
| hsa-miR-1227-3p | AP000343.2 |  | lncRNA |
| hsa-miR-485-5p | RP11-384K6.6 |  | lncRNA |
| hsa-miR-18a-3p | LL22NC03-86G7.1 |  | lncRNA |
| hsa-miR-129-5p | RP1-283E3.8 |  | lncRNA |
| hsa-miR-149-3p | RP11-630C16.2 |  | lncRNA |
| hsa-miR-129-5p | RP11-189E14.3 |  | lncRNA |
| hsa-miR-486-3p | RP4-539M6.22 |  | lncRNA |
| hsa-miR-513a-3p | AC009299.3 |  | lncRNA |
| hsa-miR-485-5p | AC138035.2 |  | lncRNA |
| hsa-miR-590-3p | LA16c-60D12.2 |  | lncRNA |
| hsa-miR-449c-5p | RP4-539M6.22 |  | lncRNA |
| hsa-miR-22-3p | AC005481.5 |  | lncRNA |
| hsa-miR-340-5p | RP11-374A4.1 |  | lncRNA |
| hsa-miR-24-3p | AC078942.1 |  | lncRNA |
| hsa-miR-939-5p | LINC00599 |  | lncRNA |
| hsa-miR-539-5p | EGFLAM-AS3 |  | lncRNA |
| hsa-miR-650 | LA16c-313D11.12 |  | lncRNA |
| hsa-miR-1207-5p | MIRLET7BHG |  | lncRNA |
| hsa-miR-1237-3p | CTC-548K16.6 |  | lncRNA |
| hsa-miR-1976 | RP11-33B1.4 |  | lncRNA |
| hsa-miR-149-3p | CTD-2369P2.8 |  | lncRNA |
| hsa-miR-765 | RP11-570L14.2 |  | lncRNA |
| hsa-miR-335-5p | RP11-1030E3.1 |  | lncRNA |
| hsa-miR-214-3p | LINC01304 |  | lncRNA |
| hsa-miR-650 | RP5-1171I10.5 |  | lncRNA |
| hsa-miR-129-5p | RP4-794I6.4 |  | lncRNA |
| hsa-miR-186-5p | SNHG14 |  | lncRNA |
| hsa-miR-590-3p | RP11-638L3.1 |  | lncRNA |
| hsa-miR-922 | LA16c-306A4.2 |  | lncRNA |
| hsa-miR-764 | RP11-830F9.6 |  | lncRNA |
| hsa-miR-940 | RP11-458F8.4 |  | lncRNA |
| hsa-miR-509-3-5p | RP3-470B24.5 |  | lncRNA |
| hsa-miR-1205 | RP3-470B24.5 |  | lncRNA |
| hsa-miR-18a-3p | RP11-54O7.17 |  | lncRNA |
| hsa-let-7f-2-3p | FAM230B |  | lncRNA |
| hsa-miR-649 | RP11-526P6.1 |  | lncRNA |
| hsa-miR-922 | RP4-539M6.22 |  | lncRNA |
| hsa-miR-15a-5p | RP11-34P13.7 |  | lncRNA |
| hsa-miR-940 | AP001476.4 |  | lncRNA |
| hsa-miR-1976 | RP11-248M19.1 |  | lncRNA |
| hsa-miR-940 | LINC00265 |  | lncRNA |
